# Costs and resource distribution of direct services for HIV in Uganda

**DOI:** 10.1136/bmjopen-2023-082062

**Published:** 2024-10-09

**Authors:** Elizabeth Ekirapa, Monica Jordan, Thuong Nong, Tozoe Elaine Marton, Hudson Balidawa, Richard Ssempala, Anthony Ssebagereka, Joseph Kagaayi, Allyala Nandakumar, Ryan K McBain

**Affiliations:** 1 Makerere University School of Public Health, Kampala, Uganda; 2 Brandeis University, Waltham, Massachusetts, USA; 3 Heller School for Social Policy and Management, Brandeis University, Waltham, Massachusetts, USA; 4 Ministry of Health, Kampala, Uganda; 5 Economic Theory and Analysis, Makerere University School of Economics, Kampala, Uganda; 6 Department of Internal Medicine, Brigham and Women's Hospital, Boston, Massachusetts, USA

**Keywords:** public health, HIV & AIDS, health economics

## Abstract

**Abstract:**

**Objective:**

In high HIV-burden countries like Uganda, financing and resource allocation for HIV services have rapidly evolved. This study aimed to employ time-driven activity-based costing (TDABC) to examine the allocation of resources and associated costs for HIV care throughout the country.

**Design:**

A cross-sectional study.

**Setting:**

This study was conducted at 31 health facilities throughout Uganda: 16 level III health centres, 10 level IV health centres and 5 district hospitals.

**Participants:**

1119 persons receiving HIV services in 2020.

**Methods:**

We conducted TDABC to quantify costs, resource consumption and duration of service provision associated with antiretroviral therapy, prevention of mother-to-child transmission, HIV counselling and testing (HCT), voluntary medical male circumcision (VMMC) and pre-exposure prophylaxis. We also quantified disparities in resource consumption according to client-level and facility-level characteristics to examine equity. Fixed-effects multivariable regression analyses were employed to inspect factors associated with service costs and provider-client interaction time.

**Results:**

The mean cost of services ranged from US$8.18 per visit for HCT to US$32.28 for VMMC. In terms of disparities, those in the Western region received more provider time during visits compared with other regions (35 more minutes, p<0.001); and those receiving care at private facilities received more provider time compared with public facilities (13 more minutes, p=0.02); and those at level IV health centres received more time compared with those at level III (12 more minutes, p=0.01). Absent consumables, services for older adults (US$2.28 higher, p=0.02), those with comorbidities (US$1.44 higher, p<0.001) and those living in the Western region (US$2.88 higher, p<0.001) were more expensive compared with younger adults, those without comorbidities and those in other regions, respectively. Inclusive of consumables, services were higher-cost for individuals in wealthier households (US$0.83 higher, p=0.03) and those visiting level IV health centres (US$3.41 higher, p=0.006) compared with level III.

**Conclusions:**

Costs and resources for HIV care vary widely throughout Uganda. This variation requires careful consideration: some sources of variation may be indicative of vertical and horizontal equity within the health system, while others may be suggestive of inequities.

**STRENGTHS AND LIMITATIONS OF THIS STUDY**

- This study is a large-scale cost analysis conducted among more than 1000 patients across 31 facilities and 8 geographical regions of Uganda.
- The study examines resource allocation not just in terms of total expenditures, but also in terms of expenditures with and without inclusion of consumables and in terms of provider time spent interacting with patients.
- The methodology, time-driven activity-based costing, allowed us to measure costs and resource consumption associated with individual patients and we could therefore measure disparities across patients according to socio-demographic characteristics.
- It is open to interpretation whether greater resource allocation to some types of clients versus others is preferable or not preferable; to some extent, it depends on the clinical judgement of experts.
- We were unable to gather information on the individual consumables expended for each patient.

## Introduction

 Uganda has one of the largest HIV-related disease burdens in sub-Saharan Africa.^1^An estimated 1.4 million individuals are living with HIV, and there were approximately 52 000 newly infected adults and children in 2022.^2^ Uganda has made significant progress in meeting the second and third targets of the 95-95-95 initiative. For example, over 95% of adults living with HIV know their status. However, progress on the first target has slowed, remaining below 90%.^3^ Notably, the neighbouring countries of Rwanda and Tanzania have successfully achieved all 95-95-95 targets.^4^

As the Government of Uganda works towards achieving the remaining 95-95-95 targets,^5^ they have updated their guidelines on HIV prevention and care. These updates, beginning in 2017, have included establishing five differentiated antiretroviral therapy (ART) models.^6^ Guidelines were again updated in 2020 to reflect new recommendations with respect to ART use, including procedures for antiretroviral (ARV) substitutions.^7^

Endeavours to bolster HIV response in Uganda have been supported by development assistance for health (DAH) from agencies such as President’s Emergency Plan for AIDS Relief (PEPFAR) and The Global Fund.^8 9^ In fiscal year 2018/2019, DAH accounted for 83% of total HIV funding.^10^ More recently, The Global Fund has advocated for a model of co-financing—with the intention to strengthen sustainability and support countries in their scale-up of domestic financing.^11 12^ Likewise, PEPFAR launched the Sustainable Financing Initiative beginning in 2014, aiming to re-align shared fiscal responsibility for healthcare with host country governments.^1^ The Government of Uganda has responded to this need for domestic resource mobilisation by ramping up domestic spending. For example, government spending increased from US$46 million in 2021 to US$83 million in 2022.^11^

Large-scale changes in financing and HIV service delivery models raise an important question concerning how HIV services have evolved at facilities throughout the country. Barriers such as stigma, insufficient training of health workers for specialised models, as well as supply chain barriers have been identified as potential constraints.^13^ Challenges in funding flows to health facilities through budget delays, as well as limited agency among facilities in the way funds are spent,^14^ could generate administrative burdens that limit progress on attaining 95-95-95 targets.

To investigate resource consumption among facilities throughout Uganda, the Government of Uganda has participated in a new initiative, the ABC/M (Activity-Based Costing/Management) Initiative,^15^ which aims to coordinate and maximise the investments made by governments and international organisations in the fight against HIV/AIDS. ABC/M applies time-driven activity-based costing (TDABC) to quantify resource allocation and consumption at the level of individual clients and facilities—including where clients go, whom they see, what services they receive and what laboratory tests and medications are ordered and prescribed.^16 17^ As one output, ABC/M provides a detailed inventory of client and cost information. While prior costing initiatives in Uganda have provided one-off estimates of a subset of HIV services in parts of the country,^18^,^20^ ABC/M is a national assessment of a comprehensive portfolio of services at health centres and hospitals, with the intention that the standardised methodology will be replicated at routine intervals. As Uganda transitions to increased domestic financing, these consistent observations will allow donors and the government alike to improve efficiency and resource consumption.

In this study, we leverage ABC/M information to examine resource consumption for HIV services throughout Uganda, quantifying costs and cost drivers across a wide range of individuals, facilities and HIV service lines. We also examine cost data from an equity perspective, analysing disparities in resource consumption. This analysis will be used by the Government of Uganda, along with their implementation partners and funders, to gain insights on how service delivery could be refined to reach more people in a more equitable manner.

## Methods

### Setting and sample characteristics

The ABC/M Initiative is currently being implemented in Kenya, Mozambique, Namibia, Tanzania, Zambia and Uganda, with Uganda being one of the first countries to complete baseline data collection. The initiative is led by the Bureau of Global Health Security and Diplomacy/PEPFAR and United States Agency for International Development (USAID), with support from several other institutions—including The Joint United Nations Programme on AIDS (UNAIDS), The Global Fund, the Centers for Disease Control and Prevention (CDC) and the US Treasury. The Government of Uganda provides the leadership and governance through an active steering committee chaired by the Ministry of Health.

Data collection concentrated on eight districts of Uganda with the highest HIV prevalence. Among these districts, 31 health facilities were chosen: 16 level III health centres, 10 level IV health centres and 5 district hospitals. These facilities were sampled to ensure balance across several characteristics, including geography (urban vs rural), facility type (eg, hospitals or health centres) and monthly volume of clients identified with HIV (low (20–249 clients), medium (250–1249 clients), high (1250+clients)) and health facility ownership (private/public) (see table 1).

**Table 1:** Characteristics of participating HIV facilities

Facility name	Region	Urbanicity	Public/private	HIV client volume
Health centres				
Amach Health Centre	Northern	Rural	Public	High
Aromo Health Centre	Northern	Rural	Public	Medium
Bondo Health Centre	Northern	Rural	Public	Medium
Bufunda Health Centre	Western	Rural	Public	High
Bugono Health Centre	Eastern	Rural	Public	Medium
Bukoto Health Centre	Central	Rural	Public	Medium
Bulamagi Health Centre	Eastern	Rural	Public	Low
Goma Health Centre	Central	Urban	Public	Medium
Ishongororo Health Centre	Western	Rural	Public	High
Kitwe Health Centre	Western	Rural	Public	High
Kiyumba Health Centre	Central	Rural	Public	Medium
Malukhu Health Centre	Eastern	Urban	Public	Medium
Mpugwe Health Centre	Central	Rural	Public	Medium
Nakaloke Health Centre	Eastern	Rural	Public	Medium
Namakwekwe Health Centre	Eastern	Urban	Public	Medium
Namatala Health Centre	Eastern	Urban	Public	Medium
Namungalwe Health Centre	Eastern	Rural	Public	Medium
River Oli Health Centre	Northern	Urban	Public	High
Rubaare Health Centre	Western	Rural	Public	High
Ruhaama Health Centre	Western	Rural	Public	Medium
Vurra Health Centre	Northern	Rural	Public	Low
Charis Health Centre	Northern	Urban	Private/PFP	Medium
Pag Mission Health Centre	Northern	Urban	Private/PNFP	High
Lambu Health Centre	Central	Rural	Private/PNFP	Medium
Kyetume Health Centre	Central	Rural	Private/PNFP	High
Hospitals				
Iganga Hospital	Eastern	Rural	Public	High
Itojo Hospital	Western	Rural	Public	High
Munoko	Central	Urban	Public	High
Kuluva Hospital	Northern	Rural	Private/PNFP	Medium
Ibanda Hospital	Western	Rural	Private/PNFP	High
Mukono C.O.U. Hospital	Central	Urban	Private/PNFP	High

HIV client volume was defined as: low (20–249 clients with HIV per month), medium (250–1249 clients), high (1250+clients). All facilities received financial contributions from PEPFARPresident’s Emergency Plan for AIDS Relief. PFP, private for-profit. PNFP, private not-for-profit

PFPprivate for-profitPNFPprivate not-for-profit

Each facility provided a wide range of comprehensive HIV services, including routine ART, HIV counselling and testing (HCT), prevention of mother-to-child transmission (PMTCT) services, voluntary medical male circumcision (VMMC) and pre-exposure prophylaxis (PrEP). Clients receiving ART services were categorised as ‘stable’ using criteria that included: having received ART for at least 6 months, with 95% adherence in the previous 6 months; no opportunistic infections in stages 3 or 4; and a viral load below 1000 copies/mL on first-line or second-line ARVs. Clients were categorised as ‘unstable’ if they did not meet these criteria.

Individuals were eligible to participate if they were aged 18 or older and accessed HIV services at 1 of the 31 participating facilities in 2020. They were identified on first entering the facility and were asked for verbal informed consent. Based on a landscape assessment, there was an initial goal of interviewing a minimum of 10 clients per facility for each of the HIV categories. Some facilities had fewer clients than expected, resulting in more clients selected from those facilities with higher numbers. For some categories, such as PrEP and VMMC, fewer than 10 clients were interviewed, as VMMC services were mainly offered through outreach efforts, which had been discontinued during the COVID-19 pandemic. Eligible participants were identified on registration at a facility. They were then informed about the study’s objectives, presented with a consent form and those who consented were assigned a unique identifier.

### Data collection

#### TDABC

A research team from Makerere University led data collection, executing TDABC procedures in accordance with best practices to measure resource consumption of direct service delivery.^13^ First, the research team and advisor board members selected medical interventions and populations to examine. Five service lines were selected (ART, HCT, PMTCT, VMMC and PrEP), with data collection limited to those 18 years or older receiving services from 1 of the 31 facilities selected. Following this, the research team worked with clinical leaders at each facility to conduct key informant interviews about care pathways and protocols corresponding to each service. These care pathways and protocols were then inspected based on direct observation of clients at each stage of their medical visit—including the order and time spent corresponding to activities, as well as the providers, equipment and physical space involved.

Next, the research team trained research assistants to observe clients at each step of their visit. Data collectors used stopwatches to time the duration of each client step, and they also interviewed providers about tasks they performed not connected to client-provider interactions. The data collectors then gathered cost information through using electronic financial systems, facility ledgers and price lists. Administrative expenses and indirect costs were also calculated and included in this analysis.

Consumables—including medications and laboratory tests—were quantified as fixed costs among clients identified as receiving associated consumables. This was elected to simplify data collection and because the provision of ARVs and laboratory tests (eg, viral load) follow a set of standard procedures, as articulated in the Consolidated Guidelines for Prevention and Treatment of HIV and AIDS in Uganda.^21^ In future rounds of data collection, it would be preferable to collect information specific to the consumables expended on behalf of each patient. Above-site costs, such as government administrative offices and supply chain, were not available for data collectors to measure. The scope of this analysis was restricted to direct service delivery.

The practical capacity of resources was estimated using the minutes per year that resources were available to clients. Capacity cost rates were calculated by dividing the cost of each resource per year by its practical capacity over the same period in order to generate the cost per minute of each resource. Then, client-level costs were calculated by multiplying capacity costs rates by the time (in minutes) that the resource was allocated to each client, in addition to the price and quantity of consumables, aggregated across all steps over the course of care delivery. Based on Uganda’s guidelines for differentiated care,^22^ we assumed quarterly facility visits per year for a stable client and monthly visits for an unstable client, annualising costs for both. All other services were interpreted as individual encounters from the point of facility entry to exit, and cost estimates therefore reflected the total cost of care during a single facility visit.

Once clients completed their medical visits, they were invited to complete a brief interview which included questions about demographic and socioeconomic status. This component of data collection, coupled with information on facility characteristics such as the type of facility (public vs private), formed the basis of our equity analysis, in which we sought to examine disparities in the utilisation of resources according to patient-level and facility-level characteristics. We note that disparities are not, in and of themselves, indicative of a normative framework concerning equity. Rather, we aimed to describe underlying sources of variation in the allocation of resources and identify whether this variation aligned with a priori expectations about vertical and horizontal equity.^23^ For the purposes of analysis described below, need can be operationally defined as the requirement for medical and associated social support services essential to the management of HIV and other challenges that complicated treatment. From this vantage point, one might assume (for example) that—on average—individuals who are elderly and frail, who have comorbid conditions or unsuppressed viral load have greater need than individuals who are young, have no comorbidities and are virally suppressed.

### Statistical analysis

We gathered descriptive statistics on service characteristics, including frequency, quantity and distribution of costs and resources for HIV service provisions—the last part of this constituting the examination of equity in resource allocation. A fixed-effects multivariable regression model was used to explore how client-level characteristics influence service duration and cost. Fixed effects were generated for the different services provided at each health facility (eg, ART, HCT, PMTCT, VMMC and PrEP) to control for the variation in the duration, cost of service and facility characteristics (ie, region, facility type, the volume of HIV clients and facility funder). The client-level characteristics we examined in this analysis include participants’ age group (18–30, 31–50, 51–70, 71+), sex (male, female), marital status (married, unmarried), years of education, comorbidity status (yes, no) and household wealth (based on a health asset index). The household asset index was calculated based on possessions in the household (eg, whether the house had a cupboard or not) and characteristics of members of the household (eg, whether or not they owned a bank account). The asset was derived from applying a routine principal component analysis, identifying a single factor.^24^

We conducted three models with the following dependent variables, corresponding to observations at the patient level: (1) the total duration of client-provider interactions over the course of a service (in minutes); (2) the total cost of the service (in US dollars), inclusive of consumables such as medications; and (3) the total cost of the service (in US dollars), excluding consumables. We conducted all analyses using Stata V.17.0, selecting a two-tailed alpha level of 0.05.^25^

### Ethics statement

The study protocol, including data collection involving human subjects, was approved by the Uganda National Council for Science and Technology and the Health Media Lab Institutional Review Board in the USA.

### Data availability statement

Data analysed during this study are not publicly available. The data were analysed by the research team at the discretion of the Ugandan Ministry of Health.

### Patients and public involvement

This study involved the public in multiple stages of implementation. First, at the stage of design and planning, a steering committee was established to provide feedback on research aims and data collection instruments. These instruments were also piloted and adapted based on feedback from local stakeholders. The time required to participate was determined early in piloting and conveyed to participants during the informed consent process. At the stages of interpretation and knowledge translation, we also approached leaders at facilities to provide feedback on the overall data collection process and results. Participants have not been involved in the dissemination of study results; instead, we have targeted dissemination through facility administrators and clinical leaders.

## Results

### Descriptive analysis

There were 1119 participants who met the inclusion criteria and consented to participate in the study. Among them, 73% were women, although this distribution varied by service line. Average age was 32.6 years old (SD: 9.4), average years of education was 2.4 (SD: 1.5) and almost half (42%) of the sample was married (see table 2). Individuals were generally low-income: for example, 11% owned a clock, 35% were connected to the electrical grid, 17% owned a bank account and 32% owned a cupboard.

**Table 2:** Study participant characteristics–by service line

Service line	Sample size	Female (%)	Age (SD)	Married (%)	Years of edu. (SD)	Comorbid conditions* (%)
ART, stable	233	152 (65)	39.4 (10.4)	99 (42)	1.7 (1.4)	15 (6)
ART, unstable	273	173 (63)	40.4 (12.7)	108 (40)	1.8 (1.3)	18 (7)
HCT	292	195 (67)	31.0 (11.5)	107 (37)	2.3 (1.5)	17 (6)
PMTCT	285	285 (100)	29.0 (5.6)	143 (50)	2.0 (1.5)	7 (2)
PrEP	17	11 (65)	30.7 (7.9)	10 (59)	2.8 (1.7)	0 (0)
VMMC	19	0 (0)	24.9 (8.4)	4 (21)	3.6 (1.4)	0 (0)
Total	**1119**	816 (73)	32.6 (9.4)	471 (42)	2.4 (1.5)	57 (5)

* 'Comorbid condition’ represents any other medical diagnosis, including infectious and non-communicable diseases, recorded during the client encounter. ART, antiretroviral therapy; HCT, ; PMTCT, prevention of mother-to-child transmission; PrEP, pre-exposure prophylaxis; , standard deviation; VMMC, .

ARTantiretroviral therapyHCTHIV counselling and testingPMTCTprevention of mother-to-child transmissionPrEPpre-exposure prophylaxisVMMCvoluntary medical male circumcision

Table 3 offers a detailed analysis of the expenses associated with each service line, categorised into four groups: resources, space/equipment, indirect costs and consumables, as well as the yearly expenditures for ART clients, both stable and unstable (4 visits per year and 12 visits per year, respectively). The highest cost per service visit was for ART for virally suppressed individuals (stable) at US$43.43, followed by VMMC (US$32.28), ART for individuals who are not virally suppressed (US$20.79), PMTCT (US$19.34), PrEP (US$10.19) and HCT (US$8.18). The average annualised ART cost for people living with HIV is US$173.72 for stable clients and US$249.48 for unstable clients. For each service line, among the four cost categories, consumables (ie, medications, laboratories and items such as syringes and gauze) accounted for most of the total cost, ranging from 73% to 95%. This was followed by human resources (4% to 23%), indirects (<1% to 2%) and space/equipment (<1% to 1%).

**Table 3:** ART annual costs and service line costs per visit

Service line	Mean cost (SD)	% of total cost	IQR
**ART - stable** *	**US$173.72 ($92.64**)	**100.00**	**US$112.12 to US$212.64**
Human resources	US$7.60 (US$10.04)	4.37	US$2.08 to US$8.2
Space/equipment	US$0.28 (US$0.72)	0.16	US$0.04 to US$0.24
Indirects	US$0.60 (US$0.68)	0.35	US$0.20 to US$0.72
Consumables	US$165.24 (US$88.52)	95.12	na†
**ART - unstable** *	**US$249.48 (US$347.16**)	**100.00**	**US$130.68 to US$324.84**
Human resources	US$23.76 (US$39.24)	8.71	$US6.96 to US$22.44
Space/equipment	US$1.20 (US$3.12)	0.43	US$0.12 to $US0.84
Indirects	US$1.92 (US$3.00)	0.72	$0.60 to $2.28
Consumables	US$246.48 (US$321.12)	90.15	na†
**HIV counselling/testing**	**US$8.18 (US$10.15**)	**100.00**	**$US3.38 to US$5.83**
Human resources	US$1.93 (US$2.62)	23.59	US$0.58 to US$2.08
Space/equipment	US$0.09 (US$0.31)	1.10	US$0.01 to US$0.05
Indirects	US$0.16 (US$0.21)	1.96	US$0.07 to US$0.17
Consumables	US$6.00 (US$9.00)	73.35	US$2.72 to US$2.95
**PMTCT**	**US$19.34 (US$13.04**)	**100.00**	**US$11.11 to US$27.38**
Human resources	US$1.95 (US$2.75)	10.08	US$0.60 to US$2.05
Space/equipment	US$0.08 (US$0.22)	0.41	US$0.01 to US $0.06
Indirects	US$0.16 (US$0.17)	0.83	US$0.06 to US $0.18
Consumables	US$17.15 (US$11.40)	88.68	US$10.41 to US$25.16
**PrEP**	**US$10.19 (US$2.13**)	**100.00**	**US$8.98 to US$11.19**
Human resources	US$1.27 (US$0.66)	12.46	US$0.78 to US$1.75
Space/equipment	US$0.02 (US$0.02)	0.20	US$0.01 to US $0.02
Indirects	US$0.10 (US$0.05)	0.98	US$0.06 to US$0.12
Consumables	US$8.80 (US$1.81)	86.36	US$7.61 to US$10.02
**VMMC**	**US$32.28 (US$5.47**)	**100.00**	**US$28.95 to US $33.69**
Human resources	US$6.42 (US$3.49)	19.89	US$4.50 to US$8.09
Space/equipment	US$0.06 (US$0.04)	0.19	US$0.02 to US$0.08
Indirects	US$0.32 (US$0.16)	0.99	US$0.21 to US$0.42
Consumables	US$25.48 (US$5.39)	78.93	US$23.8 to US$25.16

* Annualised cost presented for ART-stable and ART-unstable. All other conditions presented as cost per encounter.

† IQR was not applicable (na) for consumables because the cost values were uniform across facilities.

ART, antiretroviral therapy; HCT, HIV counselling and testing; PMTCT, prevention of mother-to-child transmission; PrEP, pre-exposure prophylaxis; VMMCvoluntary medical male circumcision

We observed significant variability in the costs for each service line across facilities. For example, the IQR for the cost of service for ART by unstable clients was US$10.89 to US$27.07 (greater than a twofold difference). For stable clients receiving ART services, this range was US$28.03 to US$53.16. Other service lines, such as PrEP and VMMC, were more consistent, with IQRs of US$8.98 to US$11.19 and US$28.95 to US$33.69, respectively.

### Multivariable regression analyses

Multivariable regression analyses examined disparities in client-level and facility-level characteristics with respect to total costs, with and without consumables and time (see table 4). We observed that having fewer household items was a statistically significant predictor of the total service costs with consumables (β=0.83, p=0.03), as well as years of education (β=−3.96 for 1–2 years, p=0.02; β=−5.69 for 3–4 years, p<0.00; and β=−8.36 for 5+years, p=0.02). When analyses were replicated without inclusion of consumables, only 3–4 years of education was a statistically significant predictor of costs (β=−0.60, p=0.06). We also found that having comorbidities was a predictor of higher total costs (β=1.44, p<0.001), as well as age. Specifically, being younger than 30 (β=0.34, p=0.007), and older than 50 (β=0.68, p=0.04 for 51–70 years old; and β=2.28, p=0.02 for years 71 plus).

**Table 4:** Results of multivariable regressions on service costs

Characteristic	Total cost, with consumables		Total cost, without consumables		Total time	
	Coef.	P value	Coef.	P value	Coef.	P value
Gender						
Female	0.99	.0.41	0.10	0.61	1.02	0.82
Age group (years)						
31–50	0.16	0.89	0.34	0.07	9.10	0.03
51–70	1.67	0.41	0.68	0.04	13.92	0.06
71+	−4.33	0.46	2.28	0.02	11.20	0.61
Marital status						
Married	−0.25	0.81	0.27	0.12	2.31	0.56
Household Items	0.83	0.03	0.08	0.22	1.23	0.38
Comorbidities	3.46	0.13	1.44	<0.001	8.96	0.30
Formal education						
1–2 years	−3.96	0.02	−0.21	0.45	−5.52	0.38
3–4 years	−5.69	<0.001	−0.60	0.06	−15.27	0.03
5+years	−8.36	0.002	−0.51	0.25	−16.16	0.11
Region						
Eastern	−1.42	0.33	−0.01	0.97	−1.91	0.73
Northern	−5.58	<0.001	0.36	0.16	−3.57	0.54
Western	7.95	<0.001	2.88	<0.001	35.01	<0.001
Facility type						
Health centre IV	3.41	0.006	0.007	0.97	11.92	0.01
Hospital	−1.18	0.40	−0.18	0.44	−2.60	0.623
Facility type						
Private	2.21	0.15	0.24	0.34	13.47	0.02
HIV intervention						
ART (stable)	26.84	<0.001	0.04	0.89	5.71	0.39
ART (unstable)	5.92	0.001	0.18	0.52	11.57	0.07
HCT	−8.19	<0.001	0.41	0.13	18.24	<0.001
PMTCT	2.80	0.11	0.37	0.20	14.31	0.03
PrEP	−2.76	0.55	0.38	0.61	10.24	0.55
VMMC	21.38	<0.001	5.79	<0.001	86.17	<0.001

For age categories, reference group is ages 18–30; for marital status, reference group is unmarried; for household assets, comparator is a one-unit difference in magnitude of household assets; for comorbidities, reference group is adults with no comorbid conditions; for education, reference group is 0 years of formal education; for region, reference region is Ccentral; for facility type, reference type is Hhealth Centercentre III, the lowest level; for facility type (public/private) reference type is public; and for intervention, reference intervention was HIV Ttreatment (new). ART, antiretroviral therapy; Coef, coefficient; HCT, ; PMTCT, prevention of mother-to-child transmission; PrEP, pre-exposure prophylaxis; VMMC, .

ARTantiretroviral therapyCoefcoefficientHCTHIV counselling and testingPMTCTprevention of mother-to-child transmissionPrEPpre-exposure prophylaxisVMMCvoluntary medical male circumcision

While compared with HIV treatment for a new client, HIV treatment for a stable client took 27 more minutes, compared with 21 min longer for VMMC and 8 fewer minutes for HCT (p<0.001). The Western region of Uganda had a higher cost with consumables, compared with the Central region (β=7.95, p<0.001), while the Northern region was lower in cost (β=−5.58, p<0.001). Health centre IV facilities were more costly than health centre III facilities (β=3.41, p<0.001). When examining facility-level costs without the inclusion of consumables, we found that most of the costs were no longer significant. Services in the Western region remained more expensive (on average), compared with the Central region (β=2.88, p<0.001) and VMMC as a service line compared with the cost of HIV testing for a new client (β=5.79, p<0.001).

Statistically significant indicators of longer provider-client interactions included being 31–50 years old (β=9.1 additional minutes, p=0.03) and between the ages of 51–71 (β=13.9 additional minutes, p=0.06), compared with a reference group of ages 18–30. Statistically significant indicators of shorter interactions included having 3–4 years of education (β=15.27 min, p=0.03), compared with 0 years of formal education. At the facility level, facilities in the Western region had longer, on average, client-provider interactions than the Central region (β=35.01, p<0.001), as did health centre IV facilities (β=11.9, p=0.1) and private facilities (β=13.5, p=0.02), compared with health centre III facilities. Additionally, compared with HIV testing for new facilities, HCT took 18 more minutes (β=18.2, p<0.001), PMTCT 14 more minutes (β=14.3, p=0.03) and VMMC 86 more minutes (β=86.2 min, p<0.001).

## Discussion

In this study of more than 1000 clients at 31 healthcare facilities in Uganda, we found that the costs of HIV service varied widely across individuals, facilities and service lines. Our findings indicate that the average cost of service provision ranged from US$8.18 for HCT to US$32.28 for VMMC. The annualised cost of ART for stable clients averaged US$173.72, while for stable clients it amounted to US$249.48.

A majority of costs were attributable to consumables, encompassing medications and laboratory services. These accounted for over 60% of total cost. Human resources accounted for 4–23% of total cost, while indirect costs constituted 1–2% and space and equipment costs were less than 1%. This cost distribution aligns with ABC/M analyses conducted in Tanzania, as well as prior research by others in Uganda.^26^,^30^ One implication of these observations is that the Ugandan health system may benefit from strengthening the supply chain, distribution and monitoring of medications and laboratory reagents. Given the role of consumables as a major cost driver in HIV services, those consumables that are expired, damaged, misplaced or stolen have the potential to represent a large financial drain.

Our analysis also showed a pattern of time and resource consumption that reflects vertical equity within the Ugandan health system, indicating that in several instances, the pattern of time and resource consumption appeared to signal a degree of vertical equity within Uganda’s health system: individuals with greater needs receive greater resources^24^ and access higher levels of care.^31^ We observed that older clients typically consumed more resources than younger clients, and clients with comorbidities had more resources allocated to them compared with those without comorbidities. Taken together, these findings suggest the healthcare delivery system is operating in a manner that is responsive to the specific needs presented by clients.

We also observed an offsetting pattern of associations, signalling a lack of equity in several areas. For instance, on average, wealthier clients received more resources—inclusive of consumables—than less wealthy clients. Similarly, clients at private facilities received, on average, 13.5 more minutes interacting with providers and staff compared with clients receiving care at public facilities. Although there may be explanations for these observations, such as wealthier individuals may be likely to pay out of pocket for additional services and private facilities may have higher staffing ratios,^32^,^34^ they nevertheless underscore an uneven distribution of care that is not driven by need. Lastly, those receiving care in the Western region of Uganda appeared to spend more time with clinicians and receive more resources, compared with those in other regions of the country.

The results of this study present auseful juxtaposition to a recent ABC/M analysis conducted in Tanzania. Overall, we found the cost of HIV services in Tanzania was lower than in Uganda, with the exception of PMTCT services. On closer examination, the reasons for this were longer visits in Uganda compared with Tanzania, along with differences in the cost of consumables. Nevertheless, it is an interesting observation that Uganda is investing more resources per capita with less progress towards 95-95-95 targets. We also observed that both countries allocated more resources to wealthy individuals receiving care, while also tending to invest more in those with greater healthcare needs. In short, both systems are striving towards equity of resources with some degree of challenges. Lastly, we observed larger regional variations in the cost of care and resource consumption in Uganda, which is consonant with tuberculosis research that has also shown significant regional differences throughout the country.^3 35^

These findings provide the Government of Uganda with the opportunity to review occurrences of inequalities in the consumption of resources throughout the county, at client and facility levels and to consider fine-tuning investments. Initiatives aimed at improving equity in resource consumption in sub-Saharan Africa, such as the Accountability for Reasonableness framework, could provide a road map.^36 37^ In addition, given that medications are a leading cost driver, it would behove the government to consider approaches to strengthening their supply chain system. Past analyses in Uganda have found challenges with stocking of essential medications.^838^,^40^ Although ARVs are often subsidised by institutions such as PEPFAR and the Global Fund, these investments may decline over time.

We acknowledge several study limitations. First, we quantified consumables as a fixed cost, although this fixed cost was only assigned to those who received consumables in accordance with specific activities such as laboratory specimen collection or pharmacy medication dispensing. Future efforts should catalogue the medications received and laboratory tests performed for individual clients. Our data also lacked resolution in other ways: for example, were unable to disentangle provider time directed towards HIV care versus care for other conditions among those presenting with comorbidities. Second, our total sample size only contains clients for whom demographic characteristics were documented. A small percentage of clients (13%) were excluded due to missing information. While we expect data are missing at random, this is not verifiable. Third, our analysis did not incorporate programme management, community-based and above-facility expenditures. Our analysis was concerned with direct service delivery, as we did not have the resources or permissions necessary to gather cost and resource information from secondary sources. Fourth, data were collected on fewer than 20 patients for PrEP and VMMC, determined by the frequency at which these services were provided. A larger sample size would be ideal to have more certainty of cost estimates. Fifth, this study tracked clients, not providers. Tracking providers could offer useful insights into activities that are not directly oriented towards clients, such as charting. Lastly, several of the conclusions we draw are based on inference, guided by the clinical and technical expertise of local staff. For example, we expected to observe that individuals with comorbidities receive greater resources than those without comorbidities, based on the assumption that comorbidities (on average) create a more complex case study for providers.

### Conclusions

This study provides an in-depth analysis of resource consumption and costs associated with HIV service delivery throughout Uganda. In addition to cataloguing cost estimates, cost variation and cost drivers, we also examined client-level and facility-level characteristics associated with provider-client interaction duration and total costs. This represented an empirical approach to assessing equity in the context of service delivery. We identified strengths (eg, older and sicker clients appeared to receive more resources) as well as weaknesses (eg, wealthier clients and those private facilities received more resources). These findings should set the stage for dialogue about best practices and help guide strategic planning efforts.

## Data Availability

No data are available.
