# Supplementary material for: Costs and resource distribution of direct services for HIV in Uganda
Source: BMJ Open. 2024 Oct 9;14(10):e082062. doi: 10.1136/bmjopen-2023-082062 (PMC11474907; doi:10.1136/bmjopen-2023-082062)
Supplement: Supplementary file 1 [file bmjopen-2023-082062.reviewer_comments.pdf]

PEER REVIEW HISTORY

BMJ Open publishes all reviews undertaken for accepted manuscripts. Reviewers are asked to complete a checklist review form and are provided with free text boxes to elaborate on their assessment. These free text comments are reproduced below.

ARTICLE DETAILS

**TITLE (PROVISIONAL)**	Costs and Resource Distribution of Direct Services for HIV in Uganda
**AUTHORS**	McBain, Ryan; Jordan, Monica; Nong, Thuong; Marton, Tozoe; Ekirapa, Elizabeth; Balidawa, Hudson; Ssempala, Richard; Ssebagereka, Anthony; Kagaayi, Joseph; Nandakumar, Allyala

VERSION 1 - REVIEW

**REVIEWER NAME**	Ngalesoni, Frida
**REVIEWER AFFILIATION**	University of Bergen
**REVIEWER CONFLICT OF INTEREST**	None
**DATE REVIEW RETURNED**	27-Dec-2023

**GENERAL COMMENTS**	Well written paper especially in the description of the costing part of the paper.<br><br>Several methodological choices will need strong justification:<br>1. Being a TDABC costing why didn't the authors find no need to accurately estimate the consumables, medications and diagnostics instead of assuming a fixed cost? What is the implication of this huge assumption in the validity of the results given this was one of the cost drivers<br><br>2. Why did the authors ignored estimation of above service level costs and still indicate that in the title that this is "health system evaluation? This costs make a considerable part of the total cost and proper and complete costing exercise will attempt even to estimate it even through rule of thumb<br><br>3. It was interesting to read the implication of comorbidities in the cost estimation even though it was not clear the interaction of resource consumption from having these comorbidities<br><br>4. The equity part of the paper lacked methodological basis to warrant having the word equity appear in the title. Authors should consider conducting basic equity analysis and explain results backed by such analysis. As it is now it is more of speculation and implication inferences by the authors<br><br>5. In related concerns proper definition of need will be required in conducting such analysis to justify equity discussion in the paper<br><br>6. Even though authors indicate "equity" as the a significant part of the, the abstract present no equity methods, no equity related results, only equity related conclusions which seems ectopic<br><br>Other general comments

	1. Why was verbal consent and not written consent sort?<br>2. The authors indicate a national representative but data collection concentrated on eight districts of Uganda with the highest HIV prevalence. Reconsider the claim of being national representative<br>3. Isn't there defined visits for stable patients e.g. 3MMD or 6MMD? Why didn't the authors instead of assumed quarterly facility visits per year for a stable clients?<br>4. I believe good publication practice is that which involves data sharing, why won't the authors share data in alignment with that practice and to allow replication and testing the results obtained

VERSION 1 – AUTHOR RESPONSE

REVIEWER #1

1. *This is a well-written paper, especially in the description of the costing part of the paper.*

We thank the Reviewer for the kind comments.

2. *Being a TDABC costing why didn't the authors find no need to accurately estimate the consumables, medications and diagnostics instead of assuming a fixed cost? What is the implication of this assumption in the validity of the results given this was one of the cost drivers.*

We agree with the Reviewer that this is an important limitation of the study. We expected data collectors to gather patient-level information on consumables, which they (unfortunately) omitted. However, the protocols for administration of most consumables—including the provision of medications and lab tests—are standardized by the Ugandan Government. For example, initial ART regimen follows a standard protocol. Furthermore, data collectors verified overall consumption of consumables across clients, implying that the average cost per client should be accurate within our data. Lastly, we only assigned consumables to clients who were documented as receiving consumables. For example, if Client A received ARVs but Client B did not, we assumed that Client A received standard ARVs and the corresponding cost for this, whereas the cost of ARVs for Client B was $0 (because no consumables were allocated). In our revision, we have further clarified these points.

3. *Why did the authors ignore estimation of above service level costs and still indicate that in the title that this is "health system evaluation? This cost make a considerable part of the total cost and proper and complete costing exercise will attempt even to estimate it even through rule of thumb*

Thank you for this question. Above-site costs such as administrative and supply chain costs were not available to the data collection team. For example, this would have required data collectors to travel to ministerial offices and storage warehouses to gather information on additional cost and resource

inputs. We agree that this is a significant limitation, which we have further emphasized in the Discussion section. Throughout the report, including the study title, we have removed the term “health system evaluation” so that readers are not confused about the fact that we limited measurement of costs to direct service delivery.

4. *It was interesting to read the implication of comorbidities in the cost estimation even though it was not clear the interaction of resource consumption from having these comorbidities*

Thank you. We were not able to disentangle resources allocated for HIV services versus comorbidities. Doing so would require clinical judgments by data collectors as to whether a particular medication or piece of equipment corresponds to one set of clinical symptoms/disease state versus another. We now list this as a limitation. We agree that capturing comorbidities is important, as many patients present with multiple conditions and symptom sets, rather than a single condition or symptom.

5. *The equity part of the paper lacked methodological basis to warrant having the word equity appear in the title. Authors should consider conducting basic equity analysis and explain results backed by such analysis. As it is now it is more of speculation and implication inferences by the authors.*

To the best of our knowledge, equity analyses typically involve an examination of disparities in the allocation and utilization of resources and services—for example, disparities according to geography, sociodemographic characteristics, or populations with disproportionate disease burden (e.g., O’Donnell et al, Analyzing Health Equity, 2007). To our knowledge, this is also true in the context of cost and cost-effectiveness analyses (e.g., Cookson et al, Using Cost Effectiveness Analysis to Address Health Equity Concerns, 2017).

In the present study, we aimed to examine disparities in allocation of resources by sex, age, marital status, household wealth, comorbidities, educational status, region, and type of facility—including once adjustments are made for covariates. In the Introduction, Methods, and Discussion sections, we now provide more detailed information about this, so it is transparent to the reader that we are construing equity analyses from this vantage point. We agree with the Reviewer that several lines of inquiry here are inferential rather than deductive: for example, we observed that more spending is allocated to those with comorbidities that those without comorbidities and that we would expect to observe this, given the fact that service delivery is more complicated with this patient population. In our revision, we raise this in our Discussion section as a limitation.

6. *In related concerns proper definition of need will be required in conducting such analysis to justify equity discussion in the paper.*

Thank you—in the Methods section where we provide additional information on the equity lens, we provide an operational definition of this construct.

7. *Even though authors indicate "equity" as the a significant part of the, the abstract present no equity methods, no equity related results, only equity related conclusions which seems off-topic.*

In our revision, we have ensured that each section of the manuscript (including those parts flagged by the Reviewer) provide additional references to the topic of equity.

8. *Why was verbal consent and not written consent sort?*

A portion of clients were illiterate, and we therefore proposed verbal administration as a more appropriate pathway, which was approved by institutional review boards.

9. *The authors indicate a national representative but data collection concentrated on eight districts of Uganda with the highest HIV prevalence. Reconsider the claim of being national representative.*

We agree with the reviewer. We have removed the term “nationally representative” from the manuscript.

10. *Isn't there defined visits for stable patients e.g. 3MMD or 6MMD? Why didn't the authors instead of assumed quarterly facility visits per year for a stable client?*

Based on guidance provided by colleagues in the Ministry, the routine pattern of service delivery for those who have achieved viral suppression is to attend facilities quarterly (3MMD). We have clarified this in the revision.

11. *I believe good publication practice is that which involves data sharing, why won't the authors share data in alignment with that practice and to allow replication and testing the results obtained?*

We thank the Reviewer for raising this question. The data analyzed in this manuscript are owned by the Ministry of Health, not by the research team (the research team was granted permission to analyze the data). The Ministry of Health is currently in the process of deciding about public availability of the data. We have now clarified this.

VERSION 2 – REVIEW

**REVIEWER NAME**	Ngalesoni, Frida
**REVIEWER AFFILIATION**	University of Bergen

**REVIEWER CONFLICT OF INTEREST**	None
**DATE REVIEW RETURNED**	16-Jul-2024

**GENERAL COMMENTS**	None

VERSION 2 – AUTHOR RESPONSE
